# On the Significance of the Quantum Mechanical Covariance Matrix

**DOI:** 10.3390/e20070500

**Published:** 2018-06-28

**Authors:** Avishy Carmi, Eliahu Cohen

**Affiliations:** 1 Center for Quantum Information Science and Technology & Faculty of Engineering Sciences, Ben-Gurion University of the Negev, Beersheba 8410501, Israel; 2 Physics Department, Centre for Research in Photonics, University of Ottawa, Advanced Research Complex, 25 Templeton, Ottawa, K1N 6N5, Canada

**Keywords:** quantum correlations, quantum bounds, nonlocality, tsallis entropy

## Abstract

The characterization of quantum correlations, being stronger than classical, yet weaker than those appearing in non-signaling models, still poses many riddles. In this work, we show that the extent of binary correlations in a general class of nonlocal theories can be characterized by the existence of a certain covariance matrix. The set of quantum realizable two-point correlators in the bipartite case then arises from a subtle restriction on the structure of this general covariance matrix. We also identify a class of theories whose covariance has neither a quantum nor an “almost quantum” origin, but which nevertheless produce the accessible two-point quantum mechanical correlators. Our approach leads to richer Bell-type inequalities in which the extent of nonlocality is intimately related to a non-additive entropic measure. In particular, it suggests that the Tsallis entropy with parameter q=1/2 is a natural operational measure of non-classicality. Moreover, when generalizing this covariance matrix, we find novel characterizations of the quantum mechanical set of correlators in multipartite scenarios. All these predictions might be experimentally validated when adding weak measurements to the conventional Bell test (without adding postselection).

## 1. Introduction

The extent of nonlocality is commonly determined by a set of correlations. In the simplest bipartite scenario, the four two-point correlators $c_{1}$, $c_{2}$, $c_{3}$ and $c_{4}$, corresponding to the four pairs of possible outcomes of Alice and Bob, may render the theory classical, quantum, or stronger-than-quantum. In this paper, we tell the richer story provided by a certain covariance matrix presented in the next section. This matrix, which may be defined in any statistical theory, implies a bound on two-point correlators analogous to that of quantum mechanics. We thus prove that all potential theories having a covariance structure similar to that of quantum mechanics have a similar set of realizable correlators. Interestingly, this is yet less than the structure imposed by quantum mechanics and theories having almost quantum correlations [1]. These results cast light on the origin of quantum correlations; they suggest that other hypothetical theories might exist whose correlations are indistinguishable from both quantum and almost quantum correlations. In this sense, our work can be seen as part of the efforts (see, e.g., [2,3,4,5,6,7,8,9]) to achieve better qualitative and quantitative understanding of quantum nonlocality.

This paper has two main parts. The first is general and does not rely on the quantum-mechanical formalism to characterize nonlocality. The second, which builds on these general results, assumes a quantum structure to derive new bounds on bipartite and tripartite two-point correlators.

Among the preceding papers in this area, there are mainly two other works where covariance and second moment matrices, different from the ones considered here, are used for characterizing quantum mechanical correlations and probability distributions: the NPA test [3], which significantly extends the approach previously employed in [10]. We note the following primary difference between these works and the paper at hand. While the positive semi-definiteness property plays a role in both, the particular covariance here leads to the identification of fundamental relations between the entries in this matrix. These relations alone are shown to govern the set of realizable binary bipartite correlators not only in quantum mechanics but in any nonlocal theory, and to imply new tighter bounds on this set.

## 2. Covariance-Based Certificate of Nonlocality

We restrict ourselves for the moment to the Bell–CHSH [11,12] setup where two experimenters perform measurements with one of their measurement devices. Alice measures using either her device 0 or device 1, and similarly Bob measures using either his device 0 or device 1. Both Alice’s outcome $a_{i}$ when she measured using device *i* and Bob’s outcome $b_{j}$ when he measured using device *j* may either be 1 or −1. We consider the products $x_{1+i+2j}=a_{i}b_{j}$ in different experiments where Alice and Bob used the pair of devices i,j. In a local hidden variables theory, the Bell–CHSH inequality, $\left|E\left[x_{1}\right]+E\left[x_{2}\right]+E\left[x_{3}\right]-E\left[x_{4}\right]\right|\leq 2$, holds [12].

Suppose now there exists a covariance matrix underlying the products $x_{1},\ldots ,x_{4}$. This 4×4 matrix is defined as

(1) $$C\overset{\mathrm{def}}{=}M-VV^{T},$$

where M is a positive semi-definite second moment matrix whose diagonal entries all equal 1, and $V^{T}=\left[c_{1},\ldots ,c_{4}\right]$ is the vector of two-point correlators. If the product $x_{i}$ is a realization of the random variable $X_{i}$, then $M_{ij}\overset{\mathrm{def}}{=}E\left[X_{i}X_{j}\right]$ and $c_{i}\overset{\mathrm{def}}{=}E\left[X_{i}\right]$, and, if it is associated with an operator $X_{i}$ (as in quantum mechanics), then $M_{ij}\overset{\mathrm{def}}{=}\frac{1}{2}\langle \left\{X_{i},X_{j}\right\}\rangle$ and $c_{i}\overset{\mathrm{def}}{=}\langle X_{i}\rangle$, where $\left\{X_{i},X_{j}\right\}\overset{\mathrm{def}}{=}X_{i}X_{j}+X_{j}X_{i}$ is the anti-commutator. The covariance is by construction real, symmetric and positive semi-definite.

However, even without specifying how the covariance is evaluated, C⪰0 (which means hereinafter that C is positive semidefinite) may be understood as an algebraic constraint on the vector of correlators that allows a covariance matrix to be defined in the underlying theory. In particular,

(2) $$VV^{T}⪯M,$$

which geometrically means that *V* is confined to the ellipsoid described by M. For example, a theory having no constraints whatsoever on the correlators may have $M=VV^{T}$. The PR-box is one such theory. It is worth noting that, in the language of [13], the left-hand side in Equation (2) is a Fisher information matrix associated with the vector *V* of correlators.

**Figure 1:**
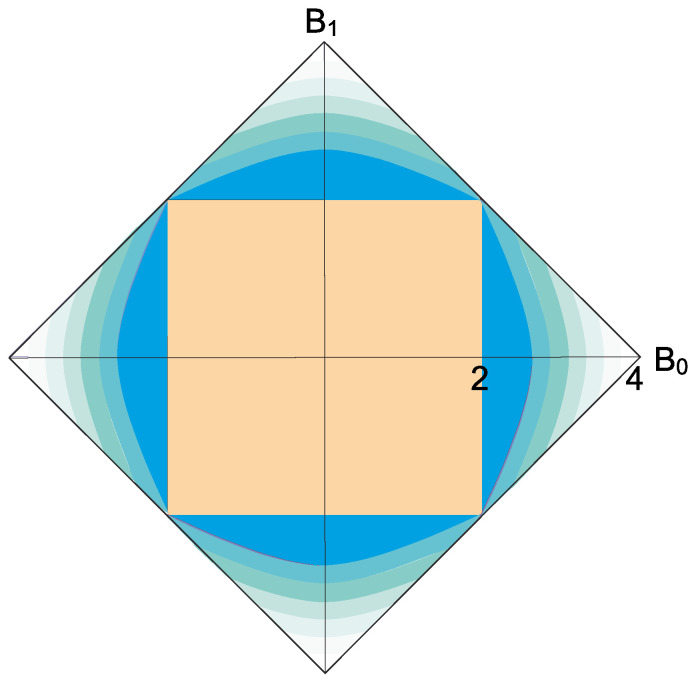
Quantum-like bounds on any statistical theory in Equation (3). The paler is the region, the larger is the difference $\left|M_{12}-M_{34}\right|$. The quantum bound on the two-point correlators, where this difference vanishes, is shown in dark blue. Classical correlators make the bounded square. In this figure, $B_{x}\overset{\mathrm{def}}{=}c_{1}+c_{2}+{\left(-1\right)}^{x}\left(c_{3}-c_{4}\right)$ is a symmetry of the Bell–CHSH parameter.

The constraint in Equation (2) leads to the following quantum-like characterization of realizable two-point correlators in any statistical theory. See Figure 1.

**Theorem** **1.** *The correlators satisfy*

(3) $$\begin{array}{c} \left|c_{1}c_{2}-c_{3}c_{4}-M_{12}+M_{34}\right|\leq \sigma_{1}\sigma_{2}+\sigma_{3}\sigma_{4}\\ \left|c_{1}c_{3}-c_{2}c_{4}-M_{13}+M_{24}\right|\leq \sigma_{1}\sigma_{3}+\sigma_{2}\sigma_{4}\\ \left|c_{2}c_{3}-c_{1}c_{4}-M_{23}+M_{14}\right|\leq \sigma_{2}\sigma_{3}+\sigma_{1}\sigma_{4}, \end{array}$$

*where $\sigma_{i}^{2}=1-c_{i}^{2}$.*

**Proof.**  The 4×4 matrix C can be partitioned into blocks as follows

(4) $$C=\left[\begin{array}{cc} D_{12} & N\\ N^{T} & D_{34}, \end{array}\right]$$

where $D_{12}$, *N* and $D_{34}$ are 2×2 matrices. Because C⪰0 so are $D_{12}⪰0$ and $D_{34}⪰0$. Therefore,

(5) $$\det\left(D_{ij}\right)=\sigma_{i}^{2}\sigma_{j}^{2}-{\left(M_{ij}-c_{i}c_{j}\right)}^{2}\geq 0,$$

namely,

(6) $$\left|M_{ij}-c_{i}c_{j}\right|\leq \sigma_{i}\sigma_{j},$$

for i,j=1,2 and i,j=3,4. This together with the triangle inequality imply

(7) $$\left|c_{1}c_{2}-c_{3}c_{4}-M_{12}+M_{34}\right|\leq \left|M_{12}-c_{1}c_{2}\right|+\left|M_{34}-c_{3}c_{4}\right|\leq \sigma_{1}\sigma_{2}+\sigma_{3}\sigma_{4}.$$

All other symmetries of this inequality in Equation (3) are obtained by swapping rows and the respective columns of C. ☐

The next corollary suggests that very little is needed to reproduce the set of quantum mechanical two-point binary correlators.

**Corollary** **1.** *The correlators vector V is realizable in quantum mechanics if and only if Equation* (2) *holds for some positive semi-definite matrix M whose diagonal entries all equal 1, and for which one of the terms, $M_{12}-M_{34}$, $M_{13}-M_{24}$, $M_{23}-M_{14}$, vanishes. In such a case,*

(8) $$\begin{array}{c} \left|c_{1}c_{2}-c_{3}c_{4}\right|\leq \sigma_{1}\sigma_{2}+\sigma_{3}\sigma_{4}\\ \left|c_{1}c_{3}-c_{2}c_{4}\right|\leq \sigma_{1}\sigma_{3}+\sigma_{2}\sigma_{4}\\ \left|c_{2}c_{3}-c_{1}c_{4}\right|\leq \sigma_{2}\sigma_{3}+\sigma_{1}\sigma_{4}. \end{array}$$

The condition in Equation (8), which from within quantum mechanics has been shown to be necessary and sufficient for quantum-realizable correlators independently by Tsirelson, Landau, and Masanes [10,14,15], is obtained here without assuming quantum mechanics, but rather from a subtle restriction on the structure of M in any statistical theory.

**Proof.**  Suppose, for example, that $M_{12}-M_{34}=0$, in which case the first inequality in Equation (3) coincides with the first inequality in Equation (8). All other symmetries of this inequality immediately follow for they are all equivalent (upon squaring, all these inequalities become identical: $2\sigma_{1}^{2}\sigma_{2}^{2}\sigma_{3}^{2}\sigma_{4}^{2}+2c_{1}c_{2}c_{3}c_{4}+2-\left(c_{1}^{2}+c_{2}^{2}+c_{3}^{2}+c_{4}^{2}\right)\geq 0$). ☐

### The Covariance in Quantum Mechanics

If all products can be factorized as $x_{1+i+2j}=a_{i}b_{j}$, where $a_{i}$ and $b_{j}$ are the local outcomes of Alice and Bob per their choices *i* and *j* (which actually amounts to the existence of local hidden variables), then Equation (3) reduces to the set of classical correlators [16]. The next theorem shows that when the products are associated with operators, a similar factorization leads to the set of quantum realizable two-point binary correlators. An important difference, then, between models of local hidden variables and quantum mechanics, is the non-commutativity of Alice’s operators, as well as the non-commutativity of Bob’s operators, which allows quantum mechanics to reach stronger correlations.

**Theorem** **2.** *Let $X_{1}\overset{\mathrm{def}}{=}A_{0}B_{0}$, $X_{2}\overset{\mathrm{def}}{=}A_{1}B_{0}$, $X_{3}\overset{\mathrm{def}}{=}A_{0}B_{1}$, and $X_{4}\overset{\mathrm{def}}{=}A_{1}B_{1}$, where the commuting operators $A_{i}$ and $B_{j}$ are self-adjoint with ±1 eigenvalues. Then, the correlations satisfy Equation* (8).

**Proof.**  The entries, $M_{12}=\langle X_{1}X_{2}+X_{2}X_{1}\rangle /2=\langle \left\{A_{0},A_{1}\right\}\rangle /2=\langle X_{3}X_{4}+X_{4}X_{3}\rangle /2=M_{34}$, and $M_{13}=\langle X_{1}X_{3}+X_{3}X_{1}\rangle /2=\langle \left\{B_{0},B_{1}\right\}\rangle /2=\langle X_{2}X_{4}+X_{4}X_{2}\rangle /2=M_{24}$. By the preceding theorem this is all that is needed to produce the quantum set of realizable two-point binary correlators. ☐

This result naturally carries over to almost quantum correlations [1] where $A_{i}B_{j}|\psi \rangle =B_{j}A_{i}|\psi \rangle$ for some states, but not necessarily all of them. Thus, in quantum theory, as well as for almost quantum correlations the matrix M has both $M_{12}-M_{34}$ and $M_{13}-M_{24}$ vanish. Interestingly, due to the preceding theorem there may exist theories, where only one of these terms vanishes, which nevertheless produce the set of quantum mechanical two-point correlators.

The quantum covariance in which $M_{12}-M_{34}=0$ and $M_{13}-M_{24}=0$ will henceforth be denoted as $C^{Q}$.

## 3. Nonlocality and Tsallis Entropy

In quantum theory and for almost quantum correlations, the extent of nonlocality may be quantified by a non-additive measure of entropy.

**Theorem** **3.** *In quantum theory, as well as for almost quantum correlations*

(9) $$\left|B\right|\leq 2+S\left(a,b\right)$$

**Figure 2:**
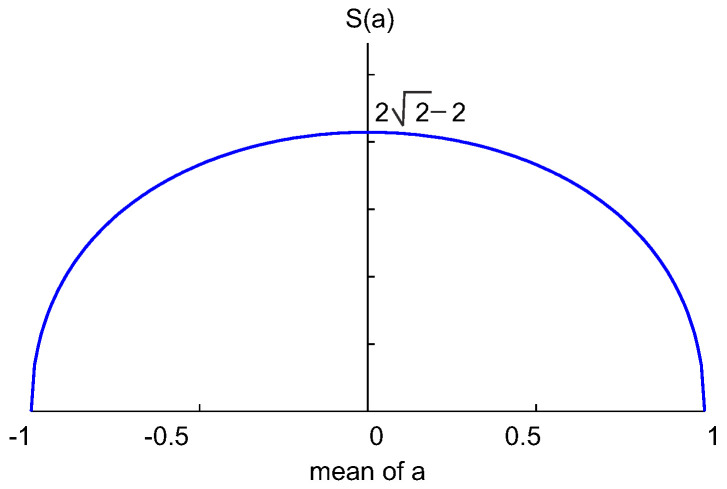
Tsallis entropy S(a) quantifies the extent of nonlocality in the Bell–CHSH experiment.

*where B is the Bell–CHSH parameter, and S(a,b) is either S(a) or S(b), the smallest among them, where S(a) and S(b) are the Tsallis entropies [17] with parameter 1/2 of a ±1-valued random variables a and b whose means are, respectively, $\langle \left\{A_{0},A_{1}\right\}\rangle /2$ and $\langle \left\{B_{0},B_{1}\right\}\rangle /2$. The right hand side in this inequality takes values between the Bell limit, 2, and the Tsirelson’s bound, $2\sqrt{2}$ (see Figure 2). The Bell bound is attained when one of the pairs, either $A_{0},A_{1}$ or $B_{0},B_{1}$, commute, and the Tsirelson’s bound is attained when both anti-commute.*

**Proof.**  The covariance matrix in Equation (1) can be partitioned as

(10) $$C^{Q}=\left[\begin{array}{cc} D_{12} & N\\ N^{T} & D_{34}, \end{array}\right]$$

where $D_{12}$, *N* and $D_{34}$ are 2×2 matrices. Because $C^{Q}⪰0$ so are $D_{12}⪰0$ and $D_{34}⪰0$. Let $g\overset{\mathrm{def}}{=}\left[1,\;\pm 1\right]$ and write

(11) $$gD_{ij}g^{T}=2\left(1\pm M_{ij}\right)-{\left(\langle X_{i}\rangle \pm \langle X_{j}\rangle \right)}^{2}\geq 0,$$

namely,

(12) $$\left|\langle X_{i}\rangle \pm \langle X_{j}\rangle \right|\leq \sqrt{2(1\pm M_{ij})},$$

for i,j=1,2 and i,j=3,4. This together with the triangleinequality yield

(13) $$\left|B\right|\leq \left|\langle X_{1}\rangle +\langle X_{2}\rangle \right|+\left|\langle X_{3}\rangle -\langle X_{4}\rangle \right|\leq \sqrt{2(1+d)}+\sqrt{2(1-d)}.$$

where $d\overset{\mathrm{def}}{=}\langle \left\{A_{0},A_{1}\right\}\rangle /2=M_{12}=M_{34}$. Let *y* be a ±1-valued random variable whose mean is *d*, i.e., p(y=±1)=(1±d)/2. The above relation can now be written as

(14) $$\left|B\right|\leq 2+S\left(a\right),$$

where the Tsallis entropy of *a* is given by

(15) $$S\left(a\right)\overset{\mathrm{def}}{=}\frac{1}{q-1}\left[1-\sum_{i=\pm 1}p{\left(a=i\right)}^{q}.\right].$$

with q=1/2. Repeating all of the above calculations for ${\tilde{C}}^{Q}$ instead of $C^{Q}$, where ${\tilde{C}}^{Q}$ is obtained by permuting the second and third columns of $C^{Q}$ and then its second and third rows, the parameter $d\overset{\mathrm{def}}{=}\langle \left\{B_{0},B_{1}\right\}\rangle /2=M_{13}=M_{24}$. ☐

This may strengthen different approaches, e.g., [18], seeking for a natural relation between uncertainty and nonlocality.

Note also that quantum and almost quantum correlations will generally have different bounds in Equation (9), depending on the pairs $A_{0},A_{1}$ and $B_{0},B_{1}$.

## 4. Verification Using Weak Measurements

The above analysis extends the ordinary Bell–CHSH experiment by introducing $d=\langle \left\{A_{0},A_{1}\right\}\rangle /2$ or $d=\langle \left\{B_{0},B_{1}\right\}\rangle /2$, i.e., a pair of local operators at either Alice’s side, Bob’s side or both. In Alice’s case, for instance, this *d* can be theoretically found once determining $A_{0}$ and $A_{1}$. However, one may question the practical feasibility of inferring it with respect to the entangled state Alice and Bob share at the same run of the ordinary Bell–CHSH experiment. We propose to measure it by employing a weak measurement [19] of the Hermitian operator $\{A_{0},A_{1}\}/2$ on Alice’s side, prior to her “strong” projective measurement. Weak measurement is known on theoretical [20] and experimental [21] grounds to asymptotically preserve entanglement, hence in the so called “weak limit” of an almost vanishing coupling constant between Alice’s qubit and the measuring pointer, the back-action of the measurement would be negligible. When accumulating large enough statistics, the expectation value *d* can be inferred with arbitrarily high accuracy. Even though each run can be thought of as measuring weakly a pre- and post-selected system, we can take the weighted sum over all weak values [19] for generating the required expectation values. The same experimental procedure can be similarly applied to any multipartite scenario.

## 5. Relation to the NPA Hierarchy

The covariance $C^{Q}$ is the Schur complement of

(16) $$N\overset{\mathrm{def}}{=}\left[\begin{array}{cc} 1 & V^{T}\\ V & C^{Q}+VV^{T} \end{array}\right]$$

Therefore, $C^{Q}⪰0$ if and only if N⪰0. This N may be viewed as a symmetrization of a Hermitian matrix Γ similar to those used in [3]. In particular,

(17) $$N=\frac{1}{2}\left(\Gamma +\Gamma^{T}\right)$$

where Γ is a submatrix in one of the levels of the NPA construction.

The symmetrization in Equation (17) allows entries whose values are otherwise inaccessible in the underlying experiment to be included in the derived bound. In fact, terms, e.g., $\langle \left\{A_{0},A_{1}\right\}\rangle /2$, which are missing from Γ, have been shown in the preceding theorem to determine the extent of nonlocality. As mentioned above, bounds involving both local and nonlocal correlations are partly motivated by a possible application of weak measurements.

## 6. Tripartite Covariance

To examine the strength and applicability of the proposed formalism, we analyze in this section and in the next one two kinds of common generalizations of the Bell–CHSH setup. First, the covariance $C^{Q}$ may be defined for any number of parties and any number of measurement devices. In the tripartite case, for example, where Alice, Bob, and Charlie each have a pair of measurement devices, the operators $X_{m}\overset{\mathrm{def}}{=}A_{i}B_{j}C_{k}$, m=1+i+2j+4k, where the commuting triplets $A_{i}$, $B_{j}$, and $C_{k}$ are self-adjoint. Here, $C^{Q}$ is an 8×8 positive semidefinite matrix.

The tripartite covariance matrix implies bounds that may be used to characterize the set of quantum realizable three-point correlators, $\langle A_{i}B_{j}C_{k}\rangle$. In this respect, the results of the preceding theorems hold for any 4×4 submatrix of any matrix obtained by permuting the columns and the respective rows of $C^{Q}$. In one case, applying the reasoning of the last theorem leads to a bound tighter than Mermin’s inequality [22]

(18) $$\left|\langle A_{0}B_{0}C_{0}\rangle +\langle A_{1}B_{1}C_{0}\rangle +\langle A_{0}B_{1}C_{1}\rangle -\langle A_{1}B_{0}C_{1}\rangle \right|\leq \sqrt{2(1+d)}+\sqrt{2(1-e)},$$

where $d=\langle \left\{A_{0}B_{0},A_{1}B_{1}\right\}\rangle /2$ and $e=\langle \left\{A_{0}B_{1},A_{1}B_{0}\right\}\rangle /2$. If both pairs, $\left(A_{0},A_{1}\right)$ and $\left(B_{0},B_{1}\right)$, commute then the right hand side in Equation (18) equals the Bell limit, 2. if, on the other hand, either one of them anti-commute, in which case d=−e, then the right hand side in this inequality reads $2\sqrt{2(1+d)}\leq 4$.

The tripartite covariance may also be composed of both two- and three-fold operators. For example, applying the first theorem to the covariance of the operators $X_{1}=A_{0}B_{j}$, $X_{2}=A_{1}B_{j}$, $X_{3}=A_{0}B_{i}C_{k}$, and $X_{4}=A_{1}B_{i}C_{k}$, yields

(19) $$\begin{array}{c} \left|\langle A_{0}B_{j}\rangle \langle A_{1}B_{j}\rangle -\langle A_{0}B_{i}C_{k}\rangle \langle A_{1}B_{i}C_{k}\rangle \right|\leq \\ \sqrt{\left(1-{\langle A_{0}B_{j}\rangle }^{2}\right)\left(1-{\langle A_{1}B_{j}\rangle }^{2}\right)}+\sqrt{\left(1-{\langle A_{0}B_{i}C_{k}\rangle }^{2}\right)\left(1-{\langle A_{1}B_{i}C_{k}\rangle }^{2}\right)}. \end{array}$$

which generalizes the bipartite inequality in [10,14,15]. The last theorem implies in this case

(20) $$\left|\langle A_{0}B_{j}\rangle +\langle A_{1}B_{j}\rangle +\langle A_{0}B_{i}C_{k}\rangle -\langle A_{1}B_{i}C_{k}\rangle \right|\leq 2+S\left(a,bc\right),$$

where the means of *a* and of bc are, respectively, $\langle \left\{A_{0},A_{1}\right\}\rangle /2$ and $\langle \left\{B_{j},B_{i}C_{k}\right\}\rangle /2$.

Consider now a tripartite covariance composed of only two-fold products, e.g., $X_{1}=A_{0}B_{j}$, $X_{2}=A_{1}B_{j}$, $X_{3}=A_{0}C_{k}$, and $X_{4}=A_{1}C_{k}$. By the last theorem

(21) $$\begin{array}{c} \left|\langle A_{0}B_{j}\rangle +\langle A_{1}B_{j}\rangle +\langle A_{0}C_{k}\rangle -\langle A_{1}C_{k}\rangle \right|\leq 2+S\left(a,bc\right)\\ \left|\langle A_{i}B_{0}\rangle +\langle A_{i}B_{1}\rangle +\langle B_{0}C_{k}\rangle -\langle B_{1}C_{k}\rangle \right|\leq 2+S\left(b,ac\right)\\ \left|\langle A_{i}C_{0}\rangle +\langle A_{i}C_{1}\rangle +\langle B_{j}C_{0}\rangle -\langle B_{j}C_{1}\rangle \right|\leq 2+S\left(c,ab\right), \end{array}$$

where the means of *a*, *b*, and *c* are, respectively, $\langle \left\{A_{0},A_{1}\right\}\rangle /2$, $\langle \left\{B_{0},B_{1}\right\}\rangle /2$, and $\langle \left\{C_{0},C_{1}\right\}\rangle /2$. Similarly, the means of ab, ac, and bc are, respectively, $\langle A_{i}B_{j}\rangle$, $\langle A_{i}C_{k}\rangle$, and $\langle B_{j}C_{k}\rangle$. These inequalities may be interpreted as follows. The first one, for example, suggests that the extent of nonlocality distributed between Alice–Bob and Alice–Charlie pairs is bounded by the local uncertainty at Alice site and also by the uncertainty underlying the Bob–Charlie link. The greater these uncertainties are, the stronger this nonlocality may get.

## 7. Further Generalization of the Covariance Matrix

The second kind of generalization refers to the natural case where Alice and Bob each have a two-level system, but now they can perform measurements in more than two incompatible bases (this is of course a very realistic scenario). For instance, when Alice and Bob may each perform three different kinds of measurements (still having ±1 outcomes), the set of products becomes $X_{k}\overset{\mathrm{def}}{=}A_{i}B_{j}$, where k=1+i+3j, and i,j∈{0,1,2}. Under the assumption of local realism one finds the following Bell inequality, $\left|B^{\prime }\right|\leq 4$, where $B^{\prime }=c_{1}+c_{2}-c_{3}+c_{4}+c_{5}+c_{6}-c_{7}+c_{8}$. This inequality is obtained from the well-studied $I_{3322}$ inequality [23] by assuming ±1 outcomes rather than 0,1 and by taking vanishing one-point correlators.

Let $C_{123}$ be the covariance of $X_{1}$, $X_{2}$, and $X_{3}$. Similarly, let $C_{456}$ and $C_{78}$ be the covariances of $X_{4}$, $X_{5}$, and $X_{6}$, and of $X_{7}$ and $X_{8}$, respectively. Because $g^{T}Cg\geq 0$, namely, $\left|g^{T}V\right|\leq \sqrt{g^{T}Mg}$, for any vector *g*, it follows that

(22) $$\begin{array}{c} \left|B^{\prime }\right|\leq \left|c_{1}+c_{2}-c_{3}\right|+\left|c_{4}+c_{5}+c_{6}\right|+\left|c_{7}-c_{8}\right|\leq \\ \sqrt{g_{++-}^{T}M_{123}g_{++-}}+\sqrt{g_{+++}^{T}M_{456}g_{+++}}+\sqrt{g_{+-}^{T}M_{78}g_{+-}}=\\ \sqrt{3+2d-2\left(e+f\right)}+\sqrt{3+2d+2\left(e+f\right)}+\sqrt{2-2d}, \end{array}$$

where $g_{+++}$, etc., are vectors whose entries are 1 or −1, depending on the specification. Here, $d=\langle \left\{A_{0},A_{1}\right\}\rangle /2$, $e=\langle \left\{A_{0},A_{2}\right\}\rangle /2$, and $f=\langle \left\{A_{1},A_{2}\right\}\rangle /2$. It is straightforward to show that the maximum of the right hand side is 5, which is obtained for e+f=0, and d=1/2. It is worth noting that this bound coincides with numerical approximations of the bound on the original $I_{3322}$ inequality in finite-dimensional Hilbert spaces [24].

## 8. Conclusions

In this paper, the analysis of a certain covariance matrix gives rise to a tight characterization of binary two-point correlators in quantum mechanics and in a general class of nonlocal theories. This formalism has further led to a natural measure of nonlocality given by the Tsallis entropy. Finally, we have discussed some generalizations of this approach and derived new bounds on tripartite two- and three-point correlators. These predictions, which often depend not only on the correlators but also on some anti-commutators might be experimentally tested with the aid of weak measurements [19], known to preserve entanglement [20,21]. That is, the nonlocal correlators can be determined as usual by performing (strong) projective measurements on the Alice and Bob sides, and at the same time weak measurements can determine the local correlators needed for the proposed bounds. As the latter involve expectation values, rather than weak values [19], summation over all postselections should be performed. Hence, all the above seems to be experimentally testable.
